# Association of carotid intima-media thickness with cardiovascular risk factors and patient outcomes in advanced chronic kidney disease: the RRI-CKD study 

**DOI:** 10.5414/CN108494

**Published:** 2015-06-03

**Authors:** Alan Hinderliter, Robin L. Padilla, Brenda W. Gillespie, Nathan W. Levin, Peter Kotanko, Margaret Kiser, Fredric Finkelstein, Sanjay Rajagopalan, Rajiv Saran

**Affiliations:** 1 Department of Internal Medicine, University of North Carolina, Chapel Hill, NC,; 2 University of Michigan-Kidney Epidemiology and Cost Center, Ann Arbor, MI,; 3 Renal Research Institute, New York, NY,; 4 Hospital of St. Raphael Yale University, New Haven, CT,; 5 Department of Cardiovascular Medicine, Ohio State University, Columbus, OH, and; 6 Department of Internal Medicine, University of Michigan, Ann Arbor, MI, USA

**Keywords:** intima-media thickness, chronic kidney disease, cardiovascular disease risk factors, cohort study

## Abstract

Background: Chronic kidney disease (CKD) is associated with accelerated atherosclerosis and an increased risk of adverse cardiovascular disease (CVD) outcomes. The relationships of intima-media thickness (IMT), a measure of subclinical atherosclerosis, with traditional and nontraditional risk factors and with adverse outcomes in CKD patients are not well-established. Methods: IMT, clinical characteristics, cardiovascular risk factors, and clinical outcomes were measured in 198 subjects from the Renal Research Institute (RRI) CKD study, a four-center prospective cohort of patients with estimated glomerular filtration rate (eGFR) ≤ 50 mL/min/1.73 m^2^ not requiring renal replacement therapy. Results: The patients averaged 61 ± 14 years of age; the mean eGFR was 29 ± 12 mL/min/1.73 m^2^. Maximum IMT was more closely associated with traditional cardiovascular risk factors, including age, diabetes, dyslipidemia, and systolic blood pressure, than with nontraditional risk factors or with eGFR. Higher values of maximum IMT were also independently associated with clinical CVD and with other markers of subclinical CVD. Maximum IMT ≥ 2.6 mm was predictive of the composite endpoint of CVD events and death (hazard ratio (HR): 5.47 (95% confidence interval (CI): 2.97 – 10.07, p < 0.0001)) but was not related to progression to end-stage renal disease (HR: 1.67 (95% CI: 0.74 – 3.76, p = 0.21)). Conclusion: In patients with advanced pre-dialysis CKD, higher maximum IMT was associated with traditional cardiovascular risk factors, CVD, and other markers of subclinical CVD and was an independent predictor of cardiovascular events and death. Additional research is needed to examine the clinical utility of IMT in the risk stratification and clinical management of patients with CKD.

## Introduction 

Chronic kidney disease (CKD) is associated with an increased risk of adverse cardiovascular disease (CVD) outcomes. In patients with kidney failure on dialysis, mortality due to CVD is 10 – 30 times that in the general population [1]. Individuals with lesser degrees of renal dysfunction are also predisposed to cardiovascular events. Several recent reports from prospective population-based studies suggest that mild-to-moderate renal insufficiency predicts cardiovascular morbidity and mortality [2, 3, 4, 5]. Indeed, patients with CKD are more likely to die of CVD than to develop renal failure [6, 7, 8]. 

In patients with CKD there is a high prevalence of traditional CVD risk factors, such as advanced age, diabetes, and hypertension [9, 10, 11, 12, 13]. Some studies, however, indicate that the relationships between estimated glomerular filtration rate (eGFR) and CVD morbidity, and mortality are independent of these characteristics [14, 15, 16]. These observations have led to the suggestion that “nontraditional” risk factors, such as inflammation, abnormal calcium and phosphorous metabolism, anemia, hyperparathyroidism, hypoalbuminemia, and albuminuria may contribute to the risk of CVD in CKD patients. 

Assessment of the intima-media thickness (IMT) of the carotid artery is a reproducible, safe, and non-invasive method of detecting subclinical atherosclerosis. Previous cross-sectional studies in cohorts without CKD have demonstrated associations between carotid IMT and both cardiovascular risk factors and the presence of CVD [17, 18, 19, 20]. Several large observational studies have also shown that carotid IMT is a predictor of coronary heart disease events that remains significant after adjustment for traditional risk factors [21, 22, 23, 24, 25, 26]. The relationship between IMT and risk for cardiovascular events in the context of a dominant risk factor such as CKD has been examined in only a few studies. 

We examined carotid IMT in patients with advanced pre-dialysis CKD enrolled in the Renal Research Institute (RRI)-CKD study to 1) assess the relationships between IMT and traditional risk factors, nontraditional risk factors, and other markers of subclinical CVD; and 2) evaluate the relationship of subclinical atherosclerosis to adverse clinical outcomes in this population. We hypothesized that IMT would be correlated with both traditional and nontraditional CVD risk factors as well as other markers of cardiac disease, and would predict both cardiovascular events and progression to end-stage renal disease. 

## Methods 

### Patient population 

The RRI-CKD study is a four-center, prospective, observational cohort study involving adult patients with moderate-to-severe CKD, not on dialysis, enrolled between 06/2000 and 02/2006 (n = 834). Details of the study have been described previously [27]. Eligibility criteria included age ≥ 18 years and estimated glomerular filtration rate (eGFR) ≤ 50 mL/min by the Cockcroft-Gault formula. When enrollment eGFR was recalculated by the abbreviated Modification of Diet in Renal Disease equation, values were greater than 50 mL/min/1.73 m^2^ in a few cases (n = 5). At enrollment and follow-up visits, data on demographic characteristics, anthropometric measures, cause of CKD, symptoms, laboratory values, and medications were collected. 

**Figure 1:**
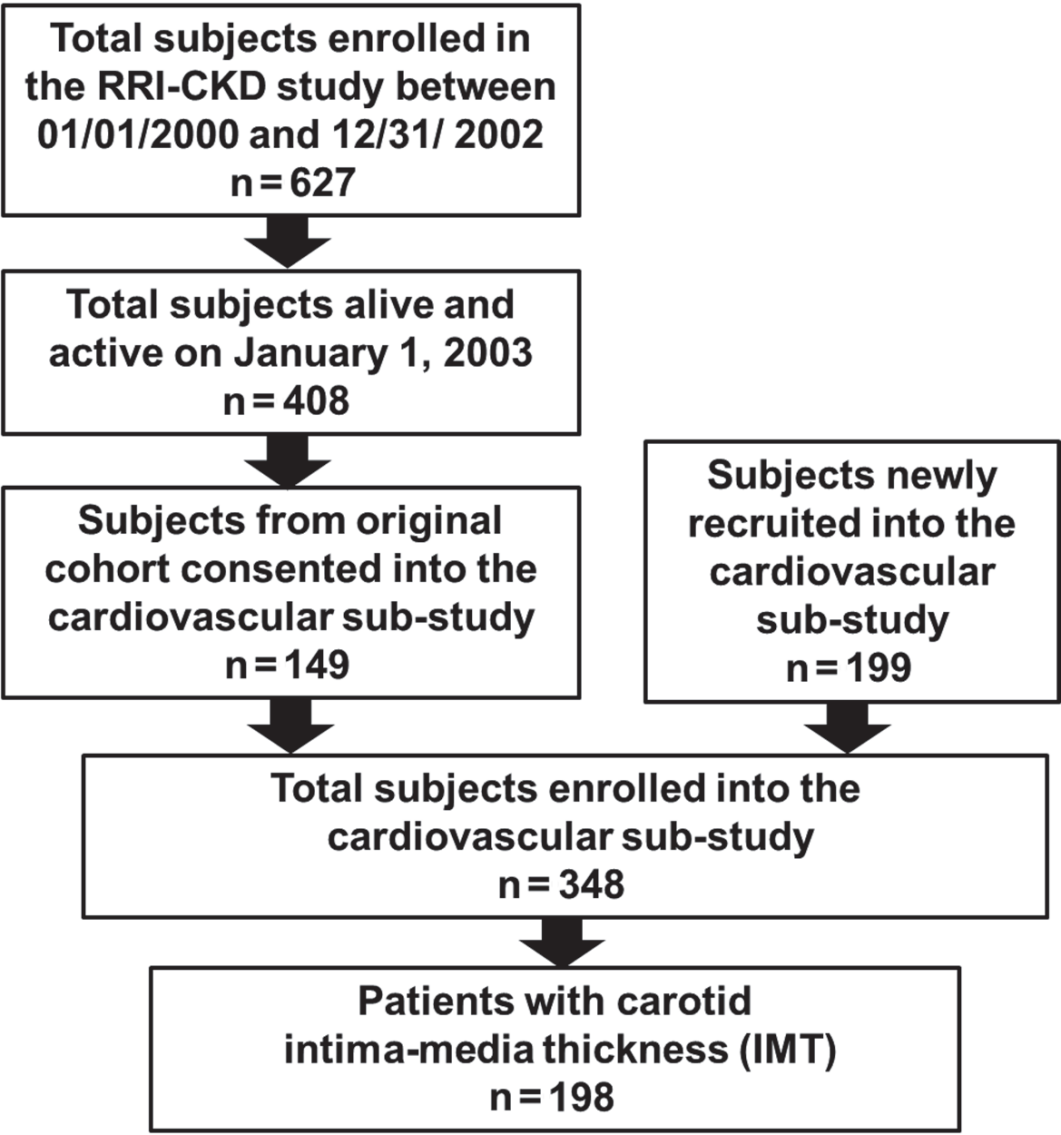
Flow-chart displaying the entry criteria into the cardiovascular sub-study and present analysis.

From January 1, 2003 onwards, a subset of individuals from the RRI-CKD cohort voluntarily consented to undergo blood, urine, and non-invasive cardiovascular studies, including measurement of carotid IMT. In addition, 199 patients were newly recruited into the CVD sub-study. While these participants were similar to the original RRI-CKD cohort participants with respect to age, diabetes, hypertension, history of CVD, race, gender, and medication use, significantly higher mean eGFR (32 vs. 24) was observed. Figure 1 displays the patient recruitment flow into this analysis. This paper is based on patients with carotid IMT data (n = 198). 

Patients who enrolled in the cardiovascular sub-study from the original RRI-CKD study (n = 149) were generally healthier than those who did not; they were younger (mean age 58 vs. 64 years), had a higher mean eGFR (27 vs. 25 mL/min/1.73 m^2^), and were less likely to have diabetes (30% vs. 42%) or a history of CVD (37% vs. 58%). The difference between the CVD sub-study and RRI-CKD participants was likely due to patient self-selection into a study that involved substantial testing and patient burden. This selection seems especially likely because the characteristics of the newly recruited CVD sub-study patients were similar to the consenters from the original RRI-CKD cohort. Finally, of the 348 participants enrolled into the cardiovascular sub-study, 150 declined measurement of IMT. These participants were similar to the 198 subjects who underwent this procedure. 

The Institutional Review Boards of the participating centers approved the protocol, and a written informed consent was obtained for all study subjects. 

### Clinical and laboratory data 

Baseline variables collected at the time of enrollment in the RRI-CKD study included the following: demographic characteristics (e.g., age, sex, and race), clinical measurements (e.g., height, weight, blood pressure, heart rate), clinical comorbidities (e.g., diabetes mellitus, hypertension, dyslipidemia, and history of tobacco use), and laboratory parameters (e.g., serum creatinine, serum calcium, serum phosphorous, serum albumin, hematocrit, intact parathyroid hormone (iPTH), lipid levels, and urine albumin-to-creatinine ratio). Blood pressure was measured in duplicate after 5 minutes of rest in the seated position using an automated, oscillometric device (Omron model HEM-412C), and the results averaged. Blood specimens were acquired after a 12-hour fast. Diabetes was defined as a fasting glucose ≥ 126 mg/dL or use of insulin or other medication for glycemic control. Hypertension was defined by a systolic BP ≥ 140 mmHg or use of antihypertensive agents. Dyslipidemia was defined as total cholesterol ≥ 240 mg/dL, LDL-cholesterol ≥ 130 mg/dL, HDL-cholesterol ≤ 40 mg/dL, or treatment with a statin. Smoking status was determined by self-report. 

### Measures of subclinical cardiovascular disease 

To insure uniformity of technique, study coordinators and sonographers at each site were trained in procedures for carotid artery ultrasound imaging and other measures of subclinical CVD at the University of Michigan data coordinating center. Images were analyzed at the core vascular laboratory at the University of Michigan. 

### Carotid intima-media thickness (IMT) 

Longitudinal images of the right and left common carotid arteries, carotid bulbs, and internal carotid arteries were acquired with a high-resolution B-mode ultrasound transducer and recorded for subsequent analysis at the core laboratory. Using electronic calipers, IMT was measured as the distance between the luminal-intima interface and the medial-adventitial interface at multiple points of the near and far walls of the distal 1 cm segments of the common carotid arteries, the carotid bulbs, and the internal carotid arteries. The mean IMT was calculated as the average of all IMT measurements; the mean of maximum IMT was calculated as the average of maximum wall thickness measurements from each region. The reproducibility of IMT measurement in the core laboratory has been described in a prior publication [28]. 

### Other measures of subclinical cardiovascular disease 

Left ventricular mass was calculated as described by Devereux et al. [29], from echocardiographic measurements of end-diastolic internal dimension and wall thicknesses. Left ventricular mass index (LVMI) was derived by correcting left ventricular mass for body surface area (LVMI = left ventricular mass/body surface area). Arterial stiffness was quantified as the carotid-femoral pulse wave velocity (PWV) using an ATCOR (version 7.0) device, as described previously [30]. Coronary calcium was measured using a 4-slice LightSpeed QXi and quantified as the Agatston score using coronary artery calcium scoring software [31]. Flow-mediated dilation of the brachial artery (FMD) was assessed by measuring changes in arterial diameter induced by reactive hyperemia and calculated as the percent change in diameter from baseline [32]. Arterial diameters at end-diastole were measured using customized software (Brachial Tools, Medical Imaging Applications, LLC, Coralville IA, USA). Measures of heart rate variability (HRV) were based on 24-hour ambulatory ECG recordings, analyzed using SyneTec Holter analysis software, version 1.20 (Ela Medical, Paris, France), as described in a prior publication [33]. 

### Clinical follow-up 

Follow-up of patients ended on December 31, 2006; all 348 patients, except 7 who were lost to follow-up, were followed for at least 10 months. All outcomes were ascertained on an ongoing basis by study coordinators, who had no knowledge of IMT results, and were based on regular review of electronic health records, direct patient contact in clinic, and periodic telephone communication. Endpoints included a composite of death or CVD events, including coronary events (myocardial infarction, coronary revascularization procedures), cerebrovascular events (stroke or transient ischemic attack), new onset heart failure, sudden cardiac death, or development of peripheral vascular disease requiring revascularization or amputation; and progression to end-stage renal disease (ESRD), defined as initiation of dialysis or pre-emptive renal transplantation. 

### Statistical methods 

Descriptive data of the study cohort are expressed as mean ± standard deviation for continuous variables (or median (interquartile range) for skewed variables) and as proportions for categorical variables. The relationships of IMT with demographic and anthropomorphic variables, clinical characteristics, traditional and non-traditional coronary risk factors, eGFR, and other measures of subclinical CVD were assessed by Pearson correlations. Multiple linear regressions were performed to assess predictors of carotid IMT, and Cox regression was used to analyze time to event outcomes. The method of best subsets was used to guide model selection process, with the R-squared selection criterion used for linear regression and the likelihood score (χ^2^) statistic criterion used for Cox regression [34]. Skewed variables were natural log transformed and a p-value < 0.05 was considered significant. Martingale residuals obtained from Cox regression models were examined to assess the correct functional form of IMT (IMT ≥ 2.6 mm). All analyses were conducted using SAS, version 9.2 (SAS Institute Inc., Cary, NC USA). 

## Results 

### Baseline patient characteristics 

Demographic and clinical characteristics of the study cohort are summarized in Table 1. The mean age of the study participants was 61 ± 14 years, with a range of 18 – 89 years. The mean eGFR was 29 ± 12 mL/min/1.73 m^2^. Nearly equal numbers had stage 3 (n = 86) and stage 4 (n = 88) CKD; 24 had stage 5 CKD. Diabetes (30%), hypertension (99%), and CVD (42%) were common comorbidities. Laboratory results and indices of subclinical CVD are shown in Table 2. 

### Associations of IMT with baseline clinical characteristics 

The median values for maximum and mean IMT were 1.21 (range: 0.48 – 4.91) mm, and 0.85 (0.44 – 2.25) mm, respectively. Analyses using maximum and mean IMT measurements yielded similar results; therefore, only those using maximum IMT are described in this manuscript. 

The correlations between IMT and eGFR, traditional risk factors, novel risk factors and other measures of CVD are summarized in Table 3. No correlation was observed between IMT and eGFR. Of traditional cardiovascular risk factors, age was most closely correlated with IMT; male gender, diabetes, current or former tobacco use, dyslipidemia, and higher systolic blood pressure (BP) were also strongly associated with increased IMT. The relationships of diabetes, dyslipidemia, and systolic BP with IMT remained significant after adjustment for age. Serum albumin was the only nontraditional risk factor associated with IMT. 

**Figure 2:**
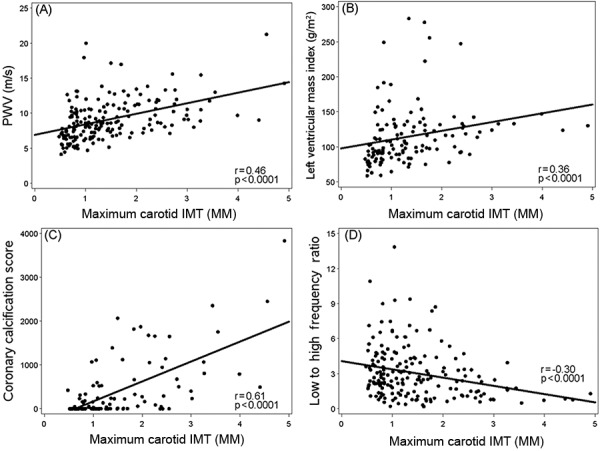
Scatter plot and least squares regression line for maximum carotid intima-media thickness (IMT) versus (A) pulse wave velocity (PWV), (B) left ventricle mass index, (C) coronary calcification score, and (D) heart rate variability (low/high frequency ratio).

Higher values of IMT were significantly associated with clinical CVD and with other markers of subclinical CVD. Patients with a clinical history of coronary artery disease, heart failure, cerebrovascular disease, and peripheral arterial disease had significantly higher values of IMT than those without known heart disease, independent of age. Figure 2 displays the positive correlations of IMT with (a) PWV, (b) LVMI, and (c) coronary calcification score, as well as (d) the negative correlation with heart rate variability (low/high frequency ratio). These relationships remained statistically significant after correcting for age. There was no significant correlation of IMT with brachial artery FMD. 

Table 4 displays results of a multivariable model examining traditional risk factors and presence of CKD as predictors of maximum IMT. Age, diabetes, dyslipidemia, systolic BP, and clinical CVD were strong independent predictors of carotid IMT. 

### Association of IMT with clinical outcomes 

Patients were followed for a median of 2.4 years following enrollment into the cardiovascular sub-study. During this time, 47 patients had at least one CVD event (n = 38) and/or died (n = 20), and 45 patients reached ESRD. 

**Figure 3:**
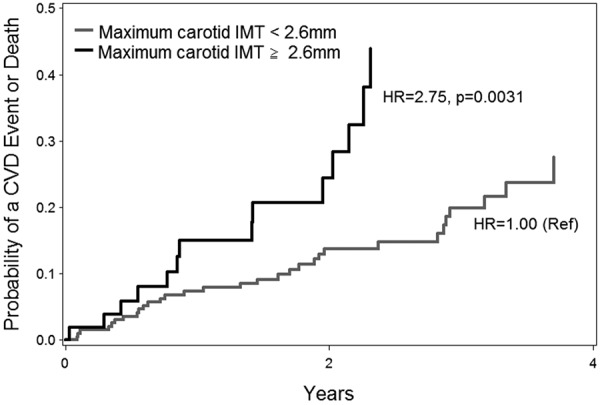
Figure 3. The cumulative probabilities of CVD events or death over time by maximum carotid intima-media thickness (IMT) above and below 2.6 mm are displayed. Martingale residuals obtained from Cox regression models were examined to assess the correct functional form of IMT (i.e., non-linear). Maximum IMT ≥ 2.6 mm was associated with a higher risk of CVD events or death, compared to patients with maximum carotid IMT < 2.6. Plotted values were calculated based on Cox regression adjusted for mean age (61), proportion of whites (0.76), proportion with diabetes (0.30), and history of CVD (0.42).

Maximum IMT ≥ 2.6 mm was associated with a composite endpoint of CVD events and death (HR: 5.47 (95% CI: 2.97 – 10.07, p < 0.0001)) but was not associated with the development of end-stage renal disease (HR: 1.67 (95% CI: 0.74 – 3.76, p = 0.21)). The survival curves shown in Figure 3 illustrate the higher probability of CVD events or death with greater maximum IMT levels. After adjustment for age, white race, diabetes, and clinical history of CVD, IMT ≥ 2.6 mm remained a strong predictor of composite endpoint (HR: 2.75 (95% CI: 1.41 – 5.38, p = 0.0031)). Maximum IMT also remained independently associated with the composite endpoint in models that included nontraditional risk factors, FMD, PWV, LVMI, or measures of HRV. 

## Discussion 

The principal findings of our study include the following: 1) patients in our cohort with advanced CKD had a high prevalence of coronary risk factors and clinical CVD; 2) IMT was more closely associated with traditional cardiovascular risk factors, such as age, diabetes, dyslipidemia, systolic BP, and tobacco use, than with nontraditional risk factors or with eGFR; 3) higher IMT was associated with clinical CVD and with other markers of subclinical disease; and 4) higher IMT predicted cardiovascular events, independent of traditional and nontraditional risk factors and prevalent CVD. 

Our finding that IMT is more closely associated with traditional risk factors than with eGFR is consistent with the results from several previous population studies [13, 35, 36]. Studies that have examined factors associated with carotid atherosclerosis in patients with advanced, pre-dialysis CKD have yielded inconsistent results. Shoji et al. [37], measured IMT in 110 non-diabetic pre-dialysis patients with CKD (serum creatinine ≥ 1.5 mg/dL), 345 non-diabetic patients with ESRD treated with maintenance hemodialysis, and 302 healthy controls. IMT was similar in patients with CKD and ESRD, and greater than in healthy controls. In the CKD group, IMT correlated significantly with age and tobacco use, and the relationship with systolic BP was of borderline significance; there was no correlation of IMT with creatinine, total cholesterol, or HDL cholesterol. Kawamato et al. [38], examined IMT in 428 men and 582 women from a single center in Japan; 49% had stage 2 CKD, and 32% had stage 3 CKD. Age, systolic blood pressure, LDL cholesterol, HDL cholesterol and eGFR were significant predictors of IMT in both genders, and eGFR was a significant independent predictor after adjusting for other risk factors. In a cohort of Chinese patients with stages 3 or 4 CKD, Szeto et al. [39], found significant associations of IMT with age, LDL-cholesterol, diabetes, and C-reactive protein, but not with systolic BP, cigarette smoking, or kidney function. Lemos et al. [40], also noted a significant correlation of IMT with C-reactive protein, as well as age and systolic BP, but not with eGFR in patients with predominantly stages 3 or 4 CKD. 

Our examination of IMT in the RRI-CKD study mirrors the results of our previously published analyses from this cohort demonstrating that changes in arterial compliance and coronary calcification are explained by conventional CVD risk factors rather than by the degree of renal dysfunction or by abnormalities in mineral metabolism. Sengstock et al. [30], examined PWV in 264 patients from the RRI-CKD cohort and 149 subjects without previously recognized CKD. Age, systolic BP, diabetes, body mass index, and heart rate were independent predictors of PWV. A statistically significant relationship of eGFR with PWV was also demonstrated, but eGFR explained less than 1% of PWV variability in models adjusting for age, systolic BP, diabetes, body mass index, and heart rate. Similarly, Dellegrottaglie et al. [31], studied determinants of coronary artery calcium in 106 patients from the RRI cardiovascular cohort who underwent multi-detector computed tomography. Coronary artery calcification was predicted by age, gender, and diabetes, but not by parameters related to renal function, including eGFR, calcium, phosphorous, i-PTH, hemoglobin, and albumin. 

Carotid IMT predicts clinical cardiovascular events, independent of traditional cardiovascular risk factors in the general population [21, 22, 23, 24, 25]. In the USE intima-media thickness (USE-IMT) global meta-analysis project using individual participant data from prospective cohort studies (n = 45,828), adjusted common carotid IMT was positively related to myocardial infarction and stroke with a hazard ratio per 0.1-mm difference of common IMT of 1.12 (95% CI 1.09 – 1.14) for women and 1.08 (95% CI 1.05 – 1.11) for men [26]. Increased IMT is also associated with adverse outcomes in dialysis patients [41, 42] and was a predictor of fatal and nonfatal cardiovascular events in subjects with stages 4 to 5 CKD enrolled in the atherosclerosis and folic acid supplementation trial [43]. The associations of IMT with prevalent CVD and with CVD events in patients with advanced pre-dialysis CKD have not been extensively studied. Adesun et al. [44], demonstrated that IMT was a predictor of self-reported CVD, with a c-statistic of 0.64, in an ancillary study of the chronic renal insufficiency cohort (CRIC) study. Szeto et al. [39] followed 203 Chinese patients with stage 3 or 4 CKD for an average of 52 months. As noted in our cohort, IMT was an independent predictor of survival free from cardiovascular events, but not of progression to ESRD. Our study extends the findings of Szeto et al. [39], by demonstrating in a multiracial cohort an association of IMT with cardiovascular events that is independent of traditional CVD risk factors. 

Previous studies of atherosclerosis in population-based cohorts have emphasized the prognostic importance of carotid plaque [21, 45]. Our analysis of outcomes using maximum IMT suggests that plaque is also a powerful predictor of adverse outcomes in patients with CKD. Mean of maximum IMT was calculated from the maximum intima-media thickness of each imaged segment, which reflects the maximum height of any plaque in that segment. Thus, high values for maximum IMT suggest the presence of plaque. A marked increase in mean maximum IMT (≥ 2.6 mm) was associated with more than a five-fold increase in the risk of cardiovascular events in our cohort, and remained significantly associated with poor outcomes after adjustment for CVD and other risk factors. 

Our study has limitations which are important to consider when interpreting the results. The absence of a clear association of IMT with eGFR may be in part due to the narrow range of values for eGFR in our subjects. It may also reflect a survival bias in this referred cohort of CKD patients; patients with more extensive atherosclerosis (and greater IMT) may have died before reaching advanced stages of CKD. Treatment with medications may also obscure relationships between risk factors and IMT. The majority of our subjects were prescribed anti-hypertensive agents, and nearly half were treated with a statin. Our cross-sectional analyses do not take into account the duration of exposure to risk factors. Although carotid IMT is independently associated with a risk of cardiovascular events, it is an imperfect measure of atherosclerosis and may not necessarily reflect disease in other vascular beds. Finally, creatinine was measured in different laboratories (at the individual sites) and GFR was estimated, not measured. 

We conclude that carotid IMT reflects clinical and subclinical CVD in patients with advanced pre-dialysis CKD and is associated with traditional cardiovascular risk factors, especially older age, hypertension, diabetes and dyslipidemia. IMT is a predictor of adverse cardiovascular events independent of traditional and novel risk factors in this population. Additional research is needed to examine the clinical utility of IMT in the risk stratification and clinical management of patients with CKD. 

## Acknowledgment 

The study was funded by the Renal Research Institute, New York, NY. We are grateful to all study coordinators and to Kerri Briesmiester, Project Manager, for training the study coordinators. 

## Conflict of interest 

The study was conducted with the support of the Michigan Clinical Research Unit, University of Michigan, funded by the NIH grant UL1RR024986. 

Dr. Nathan Levin and Dr. Peter Kotanko hold stock in Fresenius Medical Care. 

**Table 1.** Clinical characteristics at the time of non-invasive cardiovascular testing in the RRI-CKD cohort for 198 subjects with carotid intima-media thickness measurements.

	Mean ±SD or % (n)
Demographic/anthropomorphic measures	
Age (years)	61 (14)
Body mass index (kg/m^2^)	29.0 (6.3)
Male gender	53% (104)
Race	
White	76% (151)
Black	19% (37)
Other	5% (10)
Etiology of CKD	
Diabetes	26% (51)
Hypertension	53% (104)
Polycystic kidney disease	7% (14)
Interstitial renal disease	10% (20)
Glomerulonephritis	33% (65)
Other	13% (25)
Coronary risk factors	
Diabetes	30% (59)
Hypertension	99% (196)
Current smoker	9% (17)
Former smoker	40% (80)
Dyslipidemia	87% (172)
Clinical CVD	
Any clinical CVD	42% (84)
Cerebrovascular disease	11% (21)
Coronary artery disease	27% (53)
Peripheral arterial disease	15% (29)
Heart failure	19% (37)
Medications	
Diuretic	49% (97)
Renin-angiotensin-aldosterone system inhibitor	75% (148)
Beta-adrenergic receptor blocker	51% (100)
Calcium channel blocker	40% (79)
Erythropoiesis-stimulating agent	23% (45)
Statin	48% (94)
Aspirin	39% (77)

SD = standard deviation.

**Table 2.** Laboratory values and markers of subclinical cardiovascular (CV) disease at the time of non-invasive CV testing in the RRI-CKD cohort (n=198).

	Mean ± SD or median (IQR)
Indices of renal function	
Serum creatinine (mg/dL)	2.3 (1.3)
Estimated glomerular filtration rate (mL/min/1.73 m^2^)	29 ± 12
Blood urea nitrogen (mg/dL)	42.0 ± 20.1
Traditional coronary risk factors	
Total cholesterol (mg/dL)	190 ± 50
Low-density lipoprotein (mg/dL)	105 ± 39
High-density lipoprotein (mg/dL)	43 ± 17
Triglycerides (mg/dL)	148 ± 87
Systolic blood pressure (mmHg)	137 ± 24
Diastolic blood pressure (mmHg)	74 ± 14
Pulse pressure (mmHg)	63 ± 21
Heart rate (bpm)	65 ± 11
Novel risk factors	
Serum total calcium (mg/dL)	9.2 ± 0.6
Serum phosphorous (mg/dL)	3.8 ± 0.9
Serum albumin (mg/dL)	4.0 ± 0.5
Hematocrit (%)	36.2 ± 4.7
Intact parathyroid hormone (ng/mL)	117 (148)
C-reactive protein (mg/L)	2.0 (5.5)
Urine albumin/creatinine ratio (mg/g)	158 (835)
Markers of subclinical CVD	
Maximum carotid intima-medial thickness^†^ (mm)	1.21 (1.04)
Mean carotid intima-media thickness^†^ (mm)	0.85 (0.44)
Flow mediated dilation (%)	3.3 ± 4.2
Pulse wave velocity (m/s)	9.1 ± 2.9
Left ventricular mass index (g/m^2^)	103 (41)
Coronary calcification score	32 (499)
Heart rate variability: low/high frequency ratio	2.5 (2.7)
Heart rate variability: SDNN (ms)	101 (51)

SDNN = standard deviation of all normal R-R (NN) intervals; SD = standard deviation; IQR = interquartile range. ^†^Estimated CV (SD/sample mean) for the max and mean intima-medial thickness was 0.85/1.46 = 0.59, and 0.37/0.93 = 0.39, respectively.

**Table 3.** Pearson correlations of maximum carotid intima-media thickness (IMT; natural log-scale) with traditional and nontraditional risk factors and biomarkers of cardiovascular risk (n = 198).

	Pearson r	p-value
Demographic/anthropomorphic measures		
Age (years)	**0.61**	**< 0.001**
Male gender	**0.14**	**0.044**
Black race	–0.09	0.213
Body mass index (kg/m^2^)	0.05	0.445
Indices of renal function		
Serum creatinine (mg/dL)^†^	–0.02	0.829
Estimated glomerular filtration rate (mL/min/1.73 m^2^)	–0.04	0.541
Blood urea nitrogen (mg/dL)	**0.19**	**0.008**
Clinical CVD		
Any clinical CVD	**0.42**	**< 0.001**
Cerebrovascular disease	**0.16**	**0.020**
Coronary artery disease	**0.36**	**< 0.001**
Peripheral arterial disease	**0.25**	**< 0.001**
Heart failure	**0.35**	**< 0.001**
Traditional coronary risk factors		
Diabetes	**0.32**	**< 0.001**
Hypertension	0.04	0.564
Current or former smoker	**0.19**	**0.009**
Dyslipidemia	**0.20**	**0.006**
Total cholesterol (mg/dL)	–0.13	0.084
Low-density lipoprotein (mg/dL)	**–0.14**	**0.053**
High-density lipoprotein (mg/dL)	–0.01	0.901
Triglycerides (mg/dL)	0.01	0.895
Systolic blood pressure (mmHg)	**0.32**	**< 0.001**
Pulse pressure (mmHg)	**0.43**	**< 0.001**
Heart rate (bpm)	–0.01	0.903
Novel coronary risk factors		
Serum total calcium (mg/dL)	0.00	0.967
Serum phosphorous (mg/dL)	0.05	0.517
Serum albumin (mg/dL)	**–0.18**	**0.013**
Hematocrit (%)	–0.10	0.169
Intact parathyroid hormone (ng/mL)^†^	0.00	0.980
C-reactive protein (mg/L)^†^	0.11	0.126
Urine albumin/creatinine ratio (mg/g)^†^	0.03	0.683
Markers of subclinical CVD		
Flow-mediated dilation (%)	–0.06	0.397
Pulse wave velocity (m/s)	**0.46**	**< 0.001**
Left ventricular mass index (g/m^2^)^†^	**0.36**	**< 0.001**
Coronary calcification score^†^	**0.46**	**< 0.001**
Heart rate variability: low/high frequency ratio^†^	**–0.30**	**< 0.001**
Heart rate variability: SDNN (ms)^†^	0.00	0.979

SDNN = standard deviation of all normal R-R (NN) intervals; ^†^Natural log-scale.

**Table 4.** Multiple linear regression model predicting maximum carotid intima-media thickness (natural log-scale). Parameter estimates (β) and p-values are displayed (model R^2^ = 0.51, n = 198).

Variable	β	p-value
Age, per 10 years	0.20	< 0.001
White race	0.11	0.099
Diabetes	0.19	0.003
Dyslipidemia	0.24	0.005
Presence of clinical CVD	0.18	0.004
Systolic blood pressure, per 10 mmHg	0.03	0.005
